# Editorial: Emerging Functions of Septins—Volume II

**DOI:** 10.3389/fcell.2022.949824

**Published:** 2022-06-16

**Authors:** Manoj B. Menon, Matthias Gaestel

**Affiliations:** ^1^ Kusuma School of Biological Sciences, Indian Institute of Technology Delhi, New Delhi, India; ^2^ Institute for Cell Biochemistry, Hannover Medical School, Hannover, Germany

**Keywords:** septins, cytokinesis, signaling, cytoskeleton, SEPT, GTPase

The first identified members of the septin family of cytoskeletal GTPases were originally discovered in the budding yeast *Saccharomyces cerevisiae* as crucial regulators of mother-bud separation. They form a unique heteropolymeric cytoskeletal network and participate in diverse physiological functions [reviewed in (Mostowy and Cossart, 2012)]. Septin genes are conserved across eukaryotic genomes except in higher plants. In the initial decades of septin biology, research was focussed on their functions in yeast, while major breakthroughs have been achieved in the mammalian septin biology over the past two decades. Much like the intermediate filaments, the presence of heteropolymeric networks consisting of multiple septin isoforms with potential redundancy and the absence of specific inhibitors have hindered progress of septin research. In the present century, the use of mouse genetics, RNA-interference, and the advent of CRISPR/Cas9 technologies have aided in functional characterisation of septins and have uncovered isoform-specific physiological functions of septins. Septins have gained prominence as the distinct fourth component of mammalian cytoskeleton. In the first volume of the Frontiers Research topic on septins, we successfully presented contributions from across the septin field ranging from yeast cell division to human cancer and infections (Menon and Gaestel). In this second volume of the Frontiers Research topic on the emerging functions of septins, we have once again compiled a diverse collection of six reviews and four original research articles ranging from the changing models of septin oligomerisation and analyses of canonical septin functions to the cell-type specific non-canonical roles of septins in immune cells, stem cells, neurons and cancer.

The thirteen mammalian septins are classified into four groups (Septin2, Septin3, Septin6, and Septin7), named after the prominent member of the respective group. The basic repeating unit of the septin cytoskeleton is a hexamer consisting of Septin2, 6, and 7 and/or an octamer consisting of Septin2, 6, 7, and 3 group members. Until recently, the most acceptable model of the repeating unit was that of a palindromic S7-S6-S2-S2-S6-S7 hexamer and an octamer formed by addition of Septin3 group members at the termini (Sirajuddin et al., 2007; Kim et al., 2011). However, recent studies have provided evidence for revision of this model wherein the subunit organisation is inverted to give a S2-S6-S7-S7-S6-S2 core-septin hexamer (McMurray and Thorner, 2019; Mendonca et al., 2019; Soroor et al., 2021). In a thought-provoking review article, Cavini et al. provides an update on structural biology of septins with a focus on the domains and interfaces which stabilizes hexamers, octamers and the higher order filaments. While the vast majority of *in vitro*-polymerisation and GTP hydrolysis studies on mammalian septins have focussed on single septin subunits and hexamers, the focus has recently been shifted to octameric complexes (Iv et al., 2021; Soroor et al., 2021). The research article by Fischer et al. reports the biochemical characterisation of the septin octamer (S2-S6-S7-S9-S9-S7-S6-S2) and presents kinetic data on the nucleotide hydrolysis properties of human septin oligomers. Interestingly, based on the *in vitro-* polymerisation assays they propose a role for the Septin9 C-terminal region in septin filament assembly. Taking a slight deviation from the world of septin structures, Shuman and Momany review septin classification, conservation of sequence motifs in septins across kingdoms, positional orthology within heteropolymers and their evolutionary relationships. While the canonical core heteropolymers of septins are formed by the major four phylogenetic groups of septins, they present an interesting discussion on the group 5 septins, which only transiently associate with the core heteromers and are absent in the animal kingdom. Structural and functional analyses of more group 5 septins will be necessary to understand the origin and evolution of the septin family.

Septins were originally identified as proteins with role in yeast budding and the name “septin” originates from “septa” forming protein. Septins undergo cell cycle specific transitions in the budding yeast and one of the most fascinating structures formed by the septin cytoskeleton across the species is the hourglass-shaped collar formed at the mother bud-neck. In their focussed mini-review article, Marquardt et al. provide one of the best available and up-to-date summary of the molecular mechanisms driving the transition of a nascent septin ring at the bud-site to a stable hour-glass, followed by resolution into a double-ring structure facilitating budding yeast cytokinesis. Complementing this mechanistic take on the budding yeast cytokinesis is the article by Russo and Krauss which discusses the role of septins and septin remodelling in mammalian cell cytokinesis. While the central theme of septin functions in cytokinesis is established across genera and kingdoms, there are many unanswered questions regarding regulators of localized filament assembly, potential GEFs (guanine-nucleotide exchange factors), association with other cytoskeletal elements and membrane components in this process.

Despite their discovery as proteins with central role in cell division, non-dividing neuronal cells show strong expression of septins and a role for septins in neuronal morphogenesis is well established (Tada et al., 2007; Ageta-Ishihara et al., 2013). Recent work using the *Septin8* mutant mouse also indicates a role for septins in scaffolding the myelin sheath and accelerating nerve conduction (Patzig et al., 2016). One of the proposed mechanisms of septin-dependent regulation of neuronal morphogenesis is dependent on TAOK2-mediated phosphorylation of Septin7 at its C-terminal tail (Yadav et al., 2017). In their research article, Byeon et al. report the identification of phosphorylation-dependent interaction partners of Septin7 and propose a 14-3-3 dependent mechanism for septin-mediated dendritic spine maturation. This newly established link between septins and the 14-3-3 family of phosphorylation-dependent ubiquitous signalling adaptors is also expected to be relevant to other cell-types and models. Regulation of store-operated calcium signaling is another non-canonical function of the septin cytoskeleton discovered in the past decade (Sharma et al., 2013; Deb et al., 2016). In a contribution in this direction, Dhanya and Hasan investigates the role of septins in the neuronal pathogenesis associated with the deficiency of endoplasmic reticulum Ca^2+^ sensor STIM1 in mice. They report significant alleviation of the motor coordination defects in the *Stim1* knockout mouse upon the co-deletion of *Septin7* in purkinje neurons and propose septins as potential targets against neurodegenerative diseases caused by Ca^2+^ signaling defects.

Functions of septins in the cells of the hematopoietic lineages are also often linked to their membrane association and interplay with the cortical actin cytoskeleton (Gilden et al., 2012). Despite normal development of hematopoietic lineages upon pan-septin depletion in the *Septin7* knockout mice (Menon et al., 2014), septins regulate lymphocyte migration (Tooley et al., 2009) and were shown to associate with macrophage phagosomes (Huang et al., 2008), indicating clear roles in the hematopoietic system. Septins are key mediators of platelet degranulation and septin gene mutations have been associated with bleeding disorders. In an interesting mini review, Neubauer and Zieger compare septin expression and functions in platelets and endothelial cells. The recent findings from the *Septin8* knockout mice are being discussed in the context of platelet-endothelial cell interplay at the sites of vascular injury. The recent discovery from the *Septin7* knockout mice indicate a role for the Cdc42-Borg4-Septin7 axis in maintaining hematopoietic stem cell polarity and function (Kandi et al., 2021). The review by Schuster and Geiger summarises our current understanding on septin functions in stem cells and ageing. The role of septins as central mediators of asymmetric cell division from yeast to mammals is underscored in this unique review. Menon et al. reports a myeloid-specific *Septin7* conditional knockout mouse which displays the unique dichotomy of septin-dependence of cell division between monocytes and macrophages. While the development of monocytes is unaffected in the septin-deficient bone-marrow, the *in-vitro* cultivated bone-marrow derived macrophages display cytokinetic defects in the absence of Septin7. However, the multinucleated Septin7-deficient macrophages are functionally comparable to their wild-type counterparts.

Septins are crucial player in multiple stages of cell division and several studies have linked septins to cancer development (Cerveira et al., 2011; Pous et al.). However, a direct functional role for septins in tumorigenesis has not been established yet. Menon et al. describe the first *in vivo*-tumor model assessing the consequence of septin loss of function in lung cancer progression. The findings conclusively prove the role of septin-dependent proliferation in lung tumorigenesis and reconfirm the relevance of septins as targets against solid tumors.

Our understanding on the mechanisms of septin-dependent cytokinesis is constantly being revised based on findings from yeast to mammals, including the recent findings regarding the role of septins in asymmetric stem cell division. The revised order of septin subunits in the core hexamer and octamer units of mammalian septins have reignited research on redundancy and isoform-specificity of septin functions. In addition, the repertoire of cell-type specific functions of septins in neurons, hematopoietic lineages, and endothelial cells are also constantly growing. With the widespread use of CRISPR/Cas9 technology as well as septin-deficient mouse models, further physiologically relevant functions of the septin family proteins are rapidly emerging. Collectively, the articles in the second volume of this research topic, which include four original research publications from diverse aspects of mammalian septin biology, exemplarily and comprehensively summarise these advances in the field.
